# Increased C-C Chemokine Receptor 2 Gene Expression in Monocytes of Severe Obstructive Sleep Apnea Patients and under Intermittent Hypoxia

**DOI:** 10.1371/journal.pone.0113304

**Published:** 2014-11-20

**Authors:** Li-Pang Chuang, Ning-Hung Chen, Shih-Wei Lin, Ying-Ling Chang, Hsiang-Ruei Liao, Yu-Sheng Lin, I-Ju Chao, Yuling Lin, Jong-Hwei S. Pang

**Affiliations:** 1 Graduate Institute of Clinical Medical Sciences, College of Medicine, Chang Gung University, Taoyuan, Taiwan; 2 Sleep Center, Department of Pulmonary and Critical Care Medicine, Chang Gung Memorial Hospital, Taoyuan, Taiwan; 3 Department of Chinese Medicine, College of Medicine, Chang Gung University, Taoyuan, Taiwan; 4 Graduate Institute of Natural Products, College of Medicine, Chang Gung University, Taoyuan, Taiwan; 5 Division of Cardiology, Department of Internal Medicine, Chang Gung Memorial Hospital, Taipei, Taiwan; Cincinnati Children's Hospital Medical Center, United States of America

## Abstract

**Background:**

Obstructive sleep apnea (OSA) is known to be a risk factor of coronary artery disease. The chemotaxis and adhesion of monocytes to the endothelium in the early atherosclerosis is important. This study aimed to investigate the effect of intermittent hypoxia, the hallmark of OSA, on the chemotaxis and adhesion of monocytes.

**Methods:**

Peripheral blood was sampled from 54 adults enrolled for suspected OSA. RNA was prepared from the isolated monocytes for the analysis of C-C chemokine receptor 2 (CCR2). The effect of intermittent hypoxia on the regulation and function of CCR2 was investigated on THP-1 monocytic cells and monocytes. The mRNA and protein expression levels were investigated by RT/real-time PCR and western blot analysis, respectively. Transwell filter migration assay and cell adhesion assay were performed to study the chemotaxis and adhesion of monocytes.

**Results:**

Monocytic CCR2 gene expression was found to be increased in severe OSA patients and higher levels were detected after sleep. Intermittent hypoxia increased the CCR2 expression in THP-1 monocytic cells even in the presence of TNF-α and CRP. Intermittent hypoxia also promoted the MCP-1-mediated chemotaxis and adhesion of monocytes to endothelial cells. Furthermore, inhibitor for p42/44 MAPK or p38 MAPK suppressed the activation of monocytic CCR2 expression by intermittent hypoxia.

**Conclusions:**

This is the first study to demonstrate the increase of CCR2 gene expression in monocytes of severe OSA patients. Monocytic CCR2 gene expression can be induced under intermittent hypoxia which contributes to the chemotaxis and adhesion of monocytes.

## Introduction

Obstructive sleep apnea (OSA), defined as repeated episodes of obstructive apnea and hypopnea during sleep, together with symptoms of daytime sleepiness or frequently altered cardiopulmonary function, affects at least 5% of the general population [1], [2]. OSA results in intermittent hypoxia (IH) and sleep fragmentation with neurocognitive dysfunction and cardiovascular disease as the major sequelae [3]. Previous studies report that patients with sleep apnea have an increased risk of diurnal hypertension, nocturnal dysrhythmias, pulmonary hypertension, right and left ventricular failure, myocardial infarction and stroke, suggesting that sleep apnea may be one of the important risk factors of cardiovascular disorders [4], [5].

Most literatures conclude that acute coronary syndrome (ACS) would worsen OSA and vice versa, OSA would impair left ventricular function [6], [7]. Some studies further demonstrate that the morning peak distribution of ACS onset (AM 6:00∼12:00) might potentially be contributed by sleep apnea [8], [9]. Possible mechanisms responsible for the development of those cardiovascular sequelae from OSA include intermittent hypoxia and hypercapnia, exaggerated negative intrathoracic pressure and bursts of sympathetic activity, which provoke surges in blood pressure and result in endothelial cell dysfunction [10], [11]. Animal and cell culture studies have demonstrated preferential activation of inflammatory pathways by intermittent hypoxia, which is one of the integral features of OSA [12], [13].

The excessive recruitment of monocytes to the sub-endothelial space is central to the pathology of atherosclerosis. The adhesion of circulating monocytes and consequent transmigration through the vascular endothelial layer are initiated from being attracted by chemokines released from injured endothelial cells which is a crucial step in the early atherosclerosis [14]. Monocyte chemotactic protein-1 (MCP-1), abundantly present in macrophage-rich atherosclerotic plaques in human, is responsible for the recruitment, activation and differentiation of monocytes in the vascular intima space [15], [16]. Chemokine (C-C motif) receptor 2 (CCR2), one of the β-chemokine receptors characterized by seven-transmembrane domains and coupled to a GTP-binding protein is the major receptor of MCP-1. The binding of MCP-1 to CCR2 results in not only the chemotaxis of monocytes, but also the following adhesion and spreading of monocytes [17], [18]. CCR2(-/-) mice show defect in monocyte recruitment and decreased atherosclerotic lesions, indicating the important role of CCR2 in the development of atherosclerosis [19].

The plasma MCP-1 level has been found to be elevated in OSA patients that is possibly secreted not only by endothelial cells but also by monocytes from OSA patients [20]–[22]. However, there is no report regarding the CCR2 expression level or the possible regulation by intermittent hypoxia in monocytes from OSA patients. Therefore, the present study aimed to investigate the CCR2 gene expression in monocytes of OSA patients; to examine whether intermittent hypoxia can exert effect on monocytic CCR2 gene expression and its related mechanism.

## Materials and Methods

### Patients

This study was approved by the Institutional Review Board of Chang Gung Memorial Hospital (No. 100-3166B). Written informed consents were obtained from 72 patients who enrolled in this study. The inclusion criteria of participants included any adult patient (>20 years old) who was suffering from snore with the suspect of OSA diagnosis. The exclusion criteria of participants included: chronic or recent (<1 month) clinically significant infectious or inflammatory condition; asthma; trauma; invasive medical/surgical/dental procedure; recent use (<1 month) of anti-inflammatory or antibiotics drugs; coexistence of ischemic heart disease, hypertension, diabetes, hyperlipidemia, cerebrovascular disease or renal disease. After the exclusion of 18 patients (3 patients have upper airway infectious symptoms, 4 patients have high blood pressure, 2 patients have elevated blood sugar, 5 patients have hyperlipidemia and high blood pressure, 2 patients have hyperlipidemia and elevated blood sugar, 1 patient has abnormal EKG, and 1 patient has recent dental procedure within one month), 54 patients were included in this study.

### Polysomnography (PSG)

We use standard overnight PSG with the Siesta Physiological Monitoring System (Compumedics, Abbotsford, Australia) in the sleep center of our hospital with electroencephalography, electro-oculography, electromyography, and electrocardiography checks simultaneously. Ventilatory flow at the nose and mouth was measured with thermistors. Ventilatory movements of the chest and abdomen were monitored by inductive plethysmography bands. The arterial oxygen saturation (SaO_2_) was measured transcutaneously with fingertip pulse oximetry (Nonin Xpod Patient Cable Oximeter 3011). Apnea was defined as continuous cessation of airflow for more than 10 seconds and hypopnea was defined as decreasing airflow more than 30% with arousal or oxygen desaturation more than 4%. Apnea-hypopnea index (AHI) was determined by dividing the number of the total apnea/hypopnea events by the estimated hours of sleep. Adult with AHI ≦5 was considered as control, 5< AHI ≦15 as mild OSA, 15< AHI ≦30 as moderate OSA, and AHI >30 as severe OSA. Oxygen desaturation index (ODI) was determined by the number of times per hour of sleep that the blood's oxygen level drops by 4% or more from baseline. Polysomnography was performed from 10 PM to 6 AM next morning, and all the score were based on the 2010 AASM criteria.

### Blood Sampling and Monocyte Isolation

Peripheral venous blood was sampled at 6 AM after patients woke up after the performance of PSG in supine position under fasting condition. Samples were collected in heparin rinse tubes and centrifugation (3,000 rpm for 20 min) was performed within 30 min. Peripheral blood mononuclear cells isolated by Ficoll-Hypaque centrifugation were enriched for CD14+ monocytes using the autoMACS magnetic cell sorting system (Miltenyi Biotec, Bergisch Gladbach, Germany) as described previously [23]. Briefly, peripheral blood mononuclear cells were incubated with saturating concentrations of CD14 microbeads on ice for 15 min, washed, and suspended in PBS containing 2 mM EDTA and 0.5% bovine serum albumin. The cell suspension was then applied to the autoMACS separator using the positive selection program. The CD14-positive monocytes were eluted from the magnetic column and placed into 3-cm culture dishes (1×10^6^ cells per dish) containing RPMI 1640 medium supplemented with 10% (v/v) fetal bovine serum and antibiotics.

### Cell Cultures

The human monocytic leukemia cell line THP-1 was obtained from ATCC and grown in suspension culture of RPMI 1640 medium supplemented with 10% (v/v) fetal bovine serum and antibiotics. Cells were subcultured by diluting the medium with fresh growth medium in a 1∶4 ratio, and grown at 37°C in a humidified atmosphere with 5% CO_2_/95% air. Human vascular endothelial cells (HUVECs) were isolated from the vein of human umbilical cords and grown in EGM-2 provided by Clonetics (MD, USA). Cells were maintained in a humidified atmosphere with 5% CO_2_/95% air at 37°C. HUVECs were passaged 3–5 times prior to use in experiments.

### Conditions of Normoxia and Intermittent Hypoxia

Human monocytes or THP-1 cells (1×10^6^ cells/ml) were resuspended in a 5 cm culture dish containing 5 ml RPMI 1640 medium. Condition of normoxia or intermittent hypoxia (IH) was performed in a customized gas flow chamber modified from Hypo-Hyper Oxygen System (NexBioxy Inc., Taiwan). THP-1 cells or monocytes were placed in condition of normoxia (21% O_2_, 5% CO_2_, and balance N_2_) or intermittent hypoxia (6 cycles of 35 min of hypoxia [0.1% or 5% O_2_, 5% CO_2_ and balance N_2_] followed by 25 min of normoxia [21% O_2,_ 5% CO_2_ and balance N_2_] for 6 hr) and returned to normal culture condition for 18 hr before following analysis. The condition of intermittent hypoxia has used in a previously published literature and confirmed by the change of actual % O_2_ in the medium [24]. The chamber was maintained in a standard humidified incubator at 37°C. TNF-α 10 uL or CRP 10 uL are co-cultured with THP-1 cells one day before *in vivo* intermittent hypoxia study.

### RNA Extraction and RT/real-time PCR

Total cellular RNA was isolated by lysis in a guanidinium isothiocyanate buffer, followed by a single step of phenol–chloroform–isoamyl alcohol extraction. In brief, 5×10^6^ cells were lysed in 0.5 ml solution D containing 4 M guanidinium isothiocyanate, 25 mM sodium citrate (pH 7.0), 0.5% sodium sarcosine, and 0.1 M β-mercaptoethanol, with vigorous vortexing. Sequentially, 50 µl of 2 M sodium acetate (pH 4.0), 0.5 ml phenol, and 100 µl chloroform–isoamyl alcohol (49∶1, v:v) were added to the homogenate. After vortexing for 1 min, the solution was centrifuged at 12,000 rpm for 20 min at 4°C. The RNA was precipitated by the addition of 0.5 ml isopropanol and kept at −80°C for 1 h. RNA was pelleted by centrifuging the solution at 12,000 rpm for 20 min at 4°C. After the RNA pellet was rinsed in ice-cold 75% ethanol, the dry RNA was dissolved in DEPC-treated ddH_2_O. The cDNA was synthesized from total RNA using M-MLV reverse transcriptase (USB Corporation, OH, USA). Quantitative real time PCR was performed with universal cycling conditions (15 min at 95°C, followed by 40 cycles of 30 s at 95°C, 1 min at 55°C, and 30 s at 72°C). Cycle threshold (CT) values were determined by automated threshold analysis with Mx-Pro Mx3005P v4.00 software (Agilent Tech, CA, USA). PCR primers used were as follows: CCR2 forward primer, 5′-ATGCTGTCCACATCTCGTTCTCG-3′ and reverse primer, 5′-TTATAAACCAGCCGAGACTTCCTGC-3′; and GAPDH forward primer, 5′-GACCTGACCTGCCGTCTA-3′ and reverse primer, 5′-AGGAGTGGGTGTCGCTGT-3′.

### Western Blot Analysis

Cell extract was prepared by processing cells in lysis buffer containing Tris–HCl, pH 7.5, 150 mM NaCl, 1 mM EDTA, 2 mM DTT, 2 mM PMSF and 1% Triton X-100 with three times of freeze–thaw cycles and centrifugation. The 1st supernatant was used as cytosolic protein extract. The pellet was re-dissolved in lysis buffer, sonicated until the solution became clear and after centrifuging again, the 2nd supernatant was used as the membrane protein extract. The protein concentration of the cell extract was determined by Bradford assay (Bio-Rad Laboratories, CA, USA). Extracts with the same amount of proteins were separated by 10% SDS–PAGE either for coomassie blue stain or transferred onto a PVDF membrane. Membrane was incubated at 4°C in blocking solution containing 5% bovine serum albumin (BSA) in TBST for 1 h, followed by 2 h incubation in blocking solution containing appropriate dilution of primary antibody. After washing three times in TBST, the membrane was then incubated in TBST containing secondary antibody conjugated with horseradish peroxidase for 1 h. Membranes were washed three times in TBST and positive signals were developed with enhanced chemiluminescence (Amershan Pharmacia Biotech, Little Chalfont Buckinghamshire, England). The semi-quantitative measurement of the band density was calculated by using 1D Digital Analysis Software (Kodak Digital ScienceTM, Eastman Kodak, Rochester, NY). Monoclonal antibody against CCR2 was obtained from Epitomics Inc. (CA, USA). Rabbit anti-p44/p42, and goat anti-rabbit secondary antibodies conjugated with horseradish peroxidase were acquired from Cell Signaling Technology (Danvers, MA, USA).

### Transwell Filter Migration Assay

Microporous membrane (pore size, 8 µm) transwell inserts (Costar, Cambridge, MA) were used for the migration assay. THP-1 cells after normoxia or IH treatment were washed once with PBS, and 2×10^5^ cells in 200 µl RPMI were added to the upper chamber, with 400 µl RPMI containing 20 ng/ml MCP-1 in the lower chamber. Recombinant MCP-1 was purchased from R&D Systems Inc. (Minneapolis, Minnesota, USA). THP-1 cells were allowed to migrate for 1 h at 37°C in an atmosphere of 5% CO_2_/95% air and then the inserts were fixed and stained with Liu's stain. The non-migratory cells were removed before the membrane was mounted and the number of migratory cells was observed and counted under a microscope.

### Cell Adhesion Assay

THP-1 cells after normoxia or IH treatment were further activated by 20 ng/ml MCP-1 for 24 h. Recombinant MCP-1 was purchased from R&D Systems Inc. (Minneapolis, Minnesota, USA). THP-1 cells (2×10^5^) were added to the monolayer of confluent HUVECs and incubated at 37°C in a humidified 95% air/5% CO_2_ incubator for 30 min and non-adhered cells were removed by gentle washing with medium 199 twice, and the number of adhered THP-1 cells was counted using a microscope.

### Statistical Analysis

T-test was used to compare the mean value of two groups, and one-way analysis of variance (ANOVA) was used for comparing the difference of more than two groups. Correlations were examined using the Spearman rank correlation coefficient. All statistical tests were performed with the use of SPSS software (SPSS Institute, Chicago, USA). A p value of 0.05 or less is considered to indicate statistical significance, and all data was expressed as mean ± SEM.

## Results

### Increased CCR2 Gene Expression in Monocytes Isolated from Severe OSA Patients

**Figure 1:**
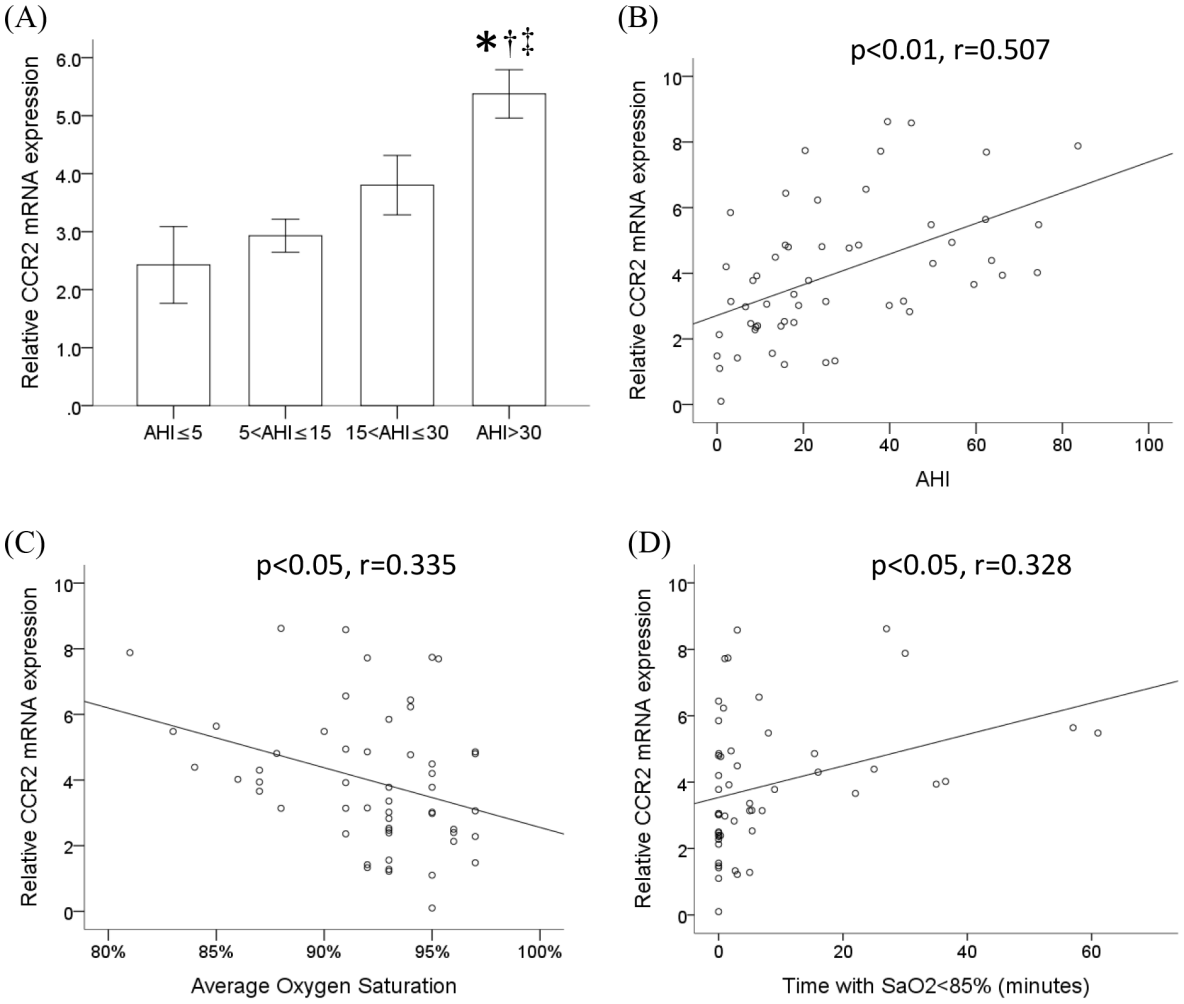
CCR2 mRNA expression significantly increased in monocytes of severe OSA patients. (A) The monocytic CCR2 mRNA expression of 54 patients from four different groups was analyzed by RT/real-time PCR. Data were means and standard errors. (mean ± SE, *: P<0.05 vs. AHI ≤5, †: P<0.05 vs 5< AHI ≤15, ‡: P<0.05 vs 15< AHI ≤30) (B) Linear regression demonstrated the positive correlation between AHI and CCR2 mRNA expression levels in monocytes (p<0.01, r = 0.507). (C) Linear regression demonstrated the negative correlation between average oxygen saturation in patients and CCR2 mRNA expression levels in monocytes (p<0.05, r = 0.335). (D) Linear regression demonstrated the positive correlation between the monocytic CCR2 mRNA expression level and the time with SaO_2_ <85% in OSA patients (p<0.05, r = 0.328).

Patients recruited in this study were divided into four groups according to the severity of OSA as indicated by apnea-hypopnea index (AHI), an index used to assess the severity of sleep apnea based on the total number of complete cessations (apnea) and partial obstructions (hypopnea) of breathing occurring per hour of sleep. Table 1 listed the demographic data of these patients in four different groups (AHI ≦5, 5< AHI ≦15, 15< AHI ≦30 and AHI >30). There was no statistic significance over age, body mass index (BMI), neck circumference and smoking status among four groups. Except sleep efficiency, the parameters of polysomnography (PSG), including AHI, ODI, mean SaO_2_, lowest SaO_2_ and time with SaO_2_ <85% showed statistic significance among these groups. Monocytes were isolated from the peripheral blood of these patients after sleep and processed for the analysis of CCR2 mRNA expression by RT/real-time PCR (Figure 1A). The monocytic CCR2 mRNA expression was found to be gradually increased along the severity of these OSA patients particularly in the group with AHI >30 which was statistically significant when compared with three other groups. Results shown in figure 1B demonstrated the positive correlation between AHI and CCR2 mRNA expression levels in monocytes (p<0.01, r = 0.507). Also, the monocytic CCR2 mRNA expression level was negatively correlated with average oxygen saturation in OSA patients (p<0.05, r = 0.335) (Figure 1C). The monocytic CCR2 mRNA expression level was positively correlated with time under condition of SaO_2_ <85% in OSA patients (p<0.05, r = 0.328) (Figure 1D).

**Table 1:** Baseline characterise and polysomnography parameters.

	Severity				
	AHI ≦5	5< AHI ≦15	15< AHI ≦30	AHI >30	
No. of subjects; (female/male)	8 (0/8)	11 (1/10)	15 (1/14)	20 (2/18)	
Age, yr	43.4±3.5	42.2±3.2	40.1±2.5	45.6±2.7	P = 0.505
BMI, kg/m^2^	24.9±1.0	24.9±1.5	26.1±0.8	28.1±1.0	P = 0.096
NC, cm	37.9±0.7	38.0±0.6	38.7±0.9	39.9±0.6	P = 0.210
Cholesterol, mg/dL	203.1±13.1	210.7±21.2	188.2±10.5	189.3±16.2	P = 0.568
Smoker/Non	3/5	4/7	6/9	9/11	P = 0.968
AHI, events/h	1.9±0.6	10.2±0.8*^a^*	20.1±1.1*^ab^*	52.4±3.4*^abc^*	P<0.001
ODI, events/h	2.7±1.4	10.0±1.2^a^	17.8±1.6^ab^	51.4±4.6^abc^	P<0.001
Sleep efficiency, %	73.3±6.6	72.2±4.8	70.1±3.1	63.7±3.2	P = 0.296
Mean SaO_2_, %	93.9±1.0	94.2±0.6	93.6±0.6	89.1±0.9*^abc^*	P<0.001
Lowest SaO_2_, %	88.6±2.2	84.8±1.2	79.1±1.6*^ab^*	74.0±2.1*^ab^*	P<0.001
Time with SaO2 <85%, minutes	0.5±0.3	0.9±0.9	2.6±2.8*^a^*	18.6±4.3*^abc^*	P<0.001

Definition of abbreviations: AHI  =  apnea-hypopnea index; BMI  =  body mass index; SaO_2_  =  oxygen saturation; NC  =  neck circumference; ODI  = 4% oxygen desaturation index. (mean ± SE, *^a^*: P<0.05 vs. AHI ≦ 5, *^b^*: P<0.05 vs. 5< AHI ≦ 15, *^c^*: P<0.05 vs. 15< AHI ≦30).

### Intermittent Hypoxia Induced the CCR2 Gene Expression in Monocytes

**Figure 2:**
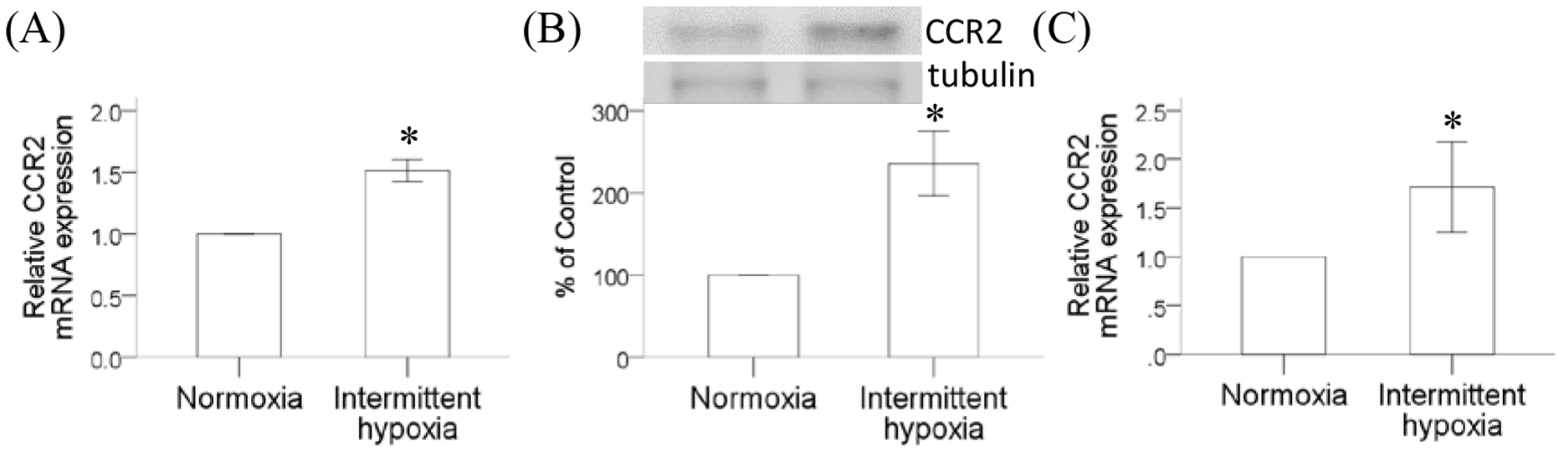
Intermittent hypoxia enhanced CCR2 gene expression in monocytes. THP-1 cells were treated with normoxia or intermittent hypoxia as described in methods. (A) RNA was isolated for the analysis of CCR2 gene expression by RT/real-time PCR. (B) Membrane proteins were prepared for western blot analysis. (C) Human peripheral monocytes were treated with the same conditions as in (A) and total RNA was isolated for the analysis of CCR2 gene expression by RT/real-time PCR. Data were present as means and standard errors from three independent experiments (mean ± SE, *: P<0.05 vs. Normoxia).

**Figure 3:**
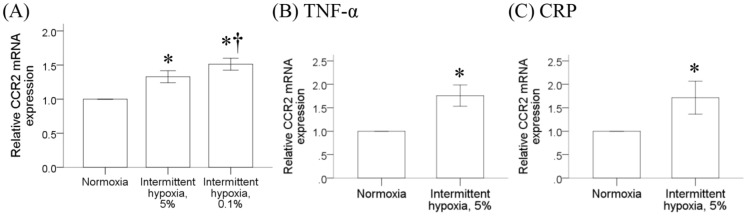
Up-regulation of monocytic CCR2 gene expression depended on the hypoxia level, but not TNF-α or CRP. (A) THP-1 cells were treated with normoxia or intermittent hypoxia with different hypoxia levels (lowest O_2_ set-point at 5% or 0.1%) as described in methods. RNA was isolated for the analysis of CCR2 mRNA expression by RT/real-time PCR. In the presence of TNF-α (B) or CRP (C), THP-1 cells were treated with intermittent hypoxia as described in methods. RNA was isolated for the analysis of CCR2 mRNA expression by RT/real-time PCR. (mean ± SE, *: P<0.05 vs. Normoxia, †: P<0.05 vs Normoxia, three independent experiments).

Since intermittent hypoxia is the hallmark of obstructive sleep apnea, we further examined the effect of intermittent hypoxia on the CCR2 expression in monocytes both at the mRNA and protein levels. Human monocytic THP-1 cells were treated with normoxia or intermittent hypoxia as described for 6 hours, and RT/real-time PCR analysis was carried out after cells were placed in incubator under normal culture condition for another 18 hours. The CCR2 mRNA expression in human monocytic THP-1 cells was significantly increased by intermittent hypoxia (Figure 2A). Result from western blot analysis comparing the membrane proteins isolated from THP-1 cells with or without intermittent hypoxia also revealed a significant increase of CCR2 protein expression induced by intermittent hypoxia (Figure 2B). Similar increase of CCR2 mRNA expression was observed when monocytes isolated from human peripheral blood were treated by intermittent hypoxia under the same condition (Figure 2C). By comparing the CCR2 mRNA expression level in monocytes under different oxygen concentration, results further demonstrated the effect of intermittent hypoxia (21%, 5% and 0.1% oxygen levels) on the CCR2 mRNA expression was dose-dependent with highest induction level in cells treated with 0.1% hypoxia (Figure 3A). In the presence of TNF-α or CRP, two inflammatory markers known to be increased in OSA patients, the CCR2 mRNA expression could be further enhanced by intermittent hypoxia, suggesting a different molecular mechanism used by intermittent hypoxia to induce the mRNA expression of CCR2 in monocytes (Figure 3B and 3C, respectively).

### Intermittent Hypoxia Enhanced Chemotaxis of Monocytes toward MCP-1

**Figure 4:**
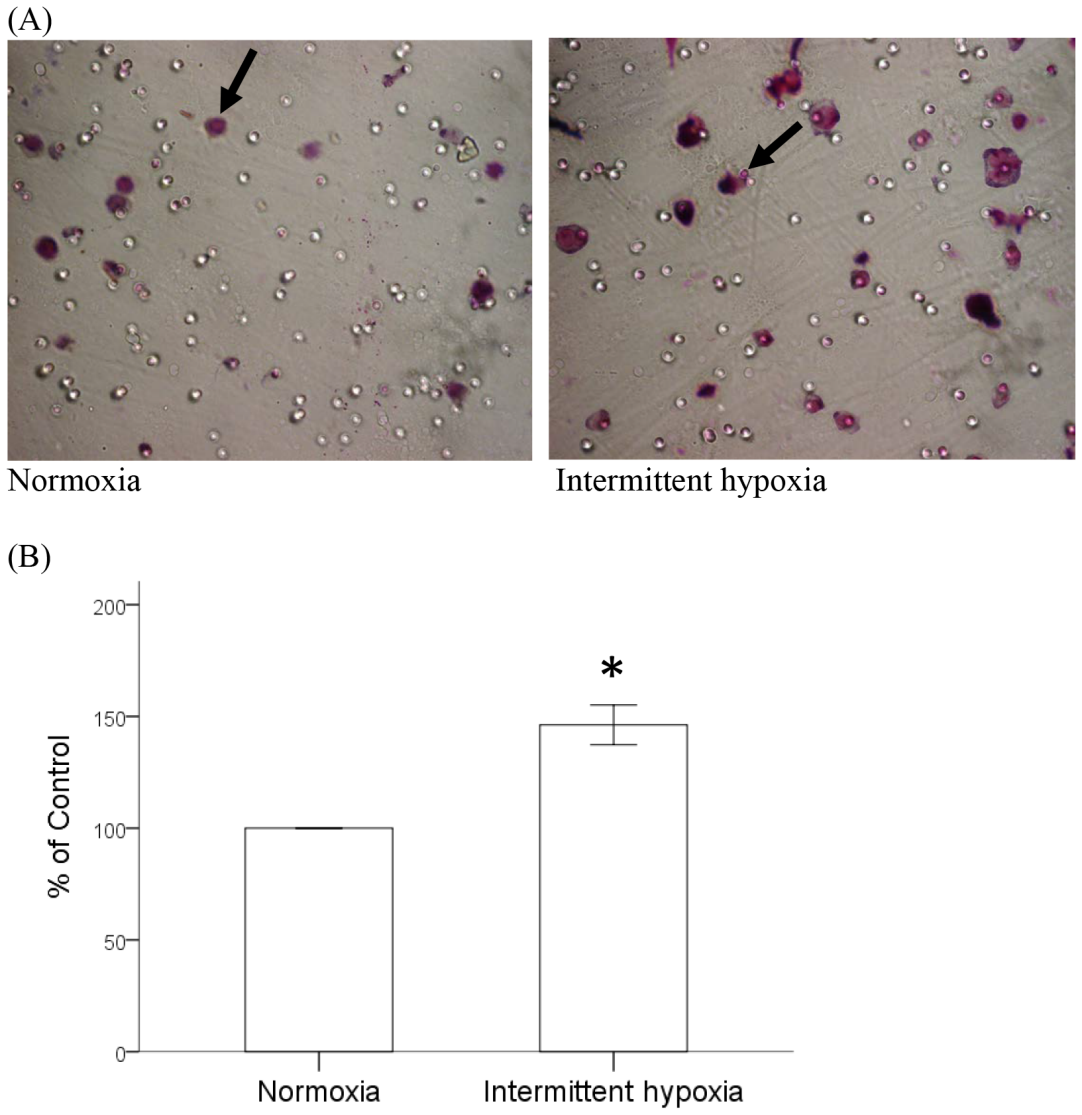
Intermittent hypoxia increased MCP-1-induced chemotaxis of monocytes. THP-1 cells were pretreated with normoxia or intermittent hypoxia as described in methods and then processed for the MCP-1-mediated chemotaxis assay. (A) Representative photos for normoxia- and intermittent hypoxia-treated THP-1 cells that migrated toward lower chamber indicated by black arrow. (B) Statistical results from three independent experiments were shown. Data were means and standard errors. (mean ± SE, *: P<0.05 vs. Normoxia).

Since the major function of CCR2 is to respond to MCP-1 and induce the chemotaxis of monocytes, we further investigated whether the increased CCR2 expression by intermittent hypoxia might affect the chemotaxis of monocytes toward MCP-1. Monocytic THP-1 cells were treated under condition of normoxia or intermittent hypoxia as described, and the chemotaxis of monocytes toward MCP-1 was analyzed by transwell migration assay for 1 hour. For the first time, we demonstrated that intermittent hypoxia could significantly enhance the chemotaxis of THP-1 cells that were attracted by MCP-1 and migrated through the transwell filter (Figure 4A and 4B).

### Intermittent Hypoxia Increased MCP-1-induced Adhesion of Monocytes to Vascular Endothelial Cells

**Figure 5:**
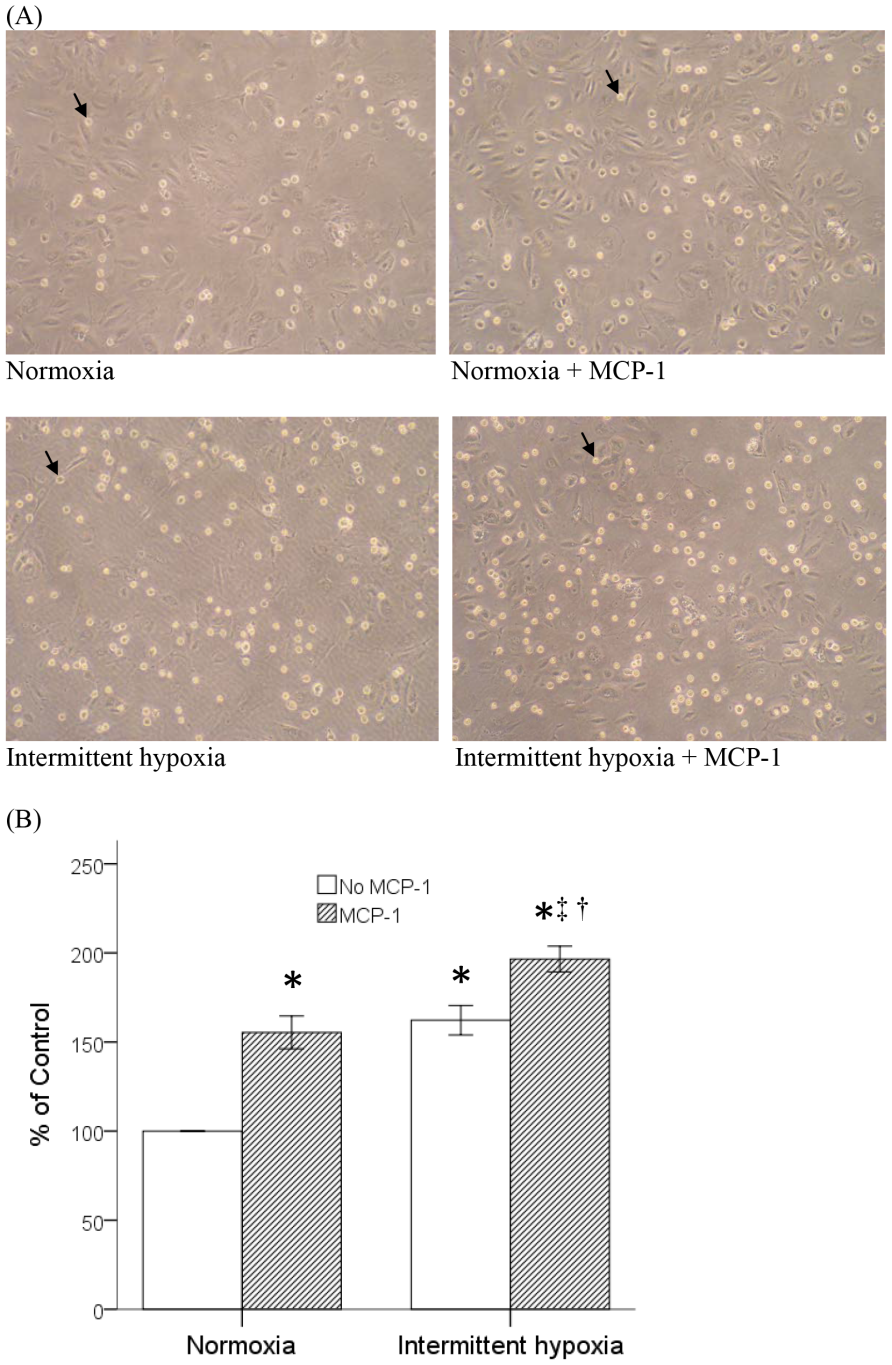
Intermittent hypoxia increased the MCP-1-enhanced adhesion of monocytes to vascular endothelial cells. THP-1 cells pretreated with normoxia or intermittent hypoxia were activated by 20 ng/ml MCP-1 for another 24 hours, and then processed for cell adhesion assay. (A) Representative photos for THP-1 cells after cell adhesion assay. Adhered cells were indicated by black arrow. (Normoxia: without any treatment, Normoxia + MCP-1: with MCP-1 stimulation only, Intermittent hypoxia: with intermittent hypoxia pretreatment only, Intermittent hypoxia + MCP-1: with intermittent hypoxia pretreatment and MCP-1 stimulation) (B) Statistical results from three independent experiments were shown. Data were means and standard errors. (mean ± SE, *: P<0.05 vs. Normoxia, †: P<0.05 vs. Normoxia + MCP-1, ‡: P<0.05 vs Intermittent hypoxia).

The activation of monocytes by MCP-1 includes the enhanced ability of adhesion to the vascular endothelial cells which also contributes to the early development of atherosclerosis [25]. We therefore examined the modulating effect of intermittent hypoxia on this MCP-1-induced adhesive activity of monocytes. THP-1 cells were pretreated with normoxia or intermittent hypoxia as described and processed for MCP-1-stimulated adhesion assay. Results demonstrated that treatment with MCP-1 or intermittent hypoxia alone enhanced the adhesion of monocytes to vascular endothelial layer and combined MCP-1 and intermittent hypoxia treatment synergistically promoted the adhesive activity of monocytes (Figure 5A and 5B).

### Intermittent hypoxia increased the CCR2 expression in monocytes through the activation of ERK and p38 MAPK signal pathways

**Figure 6:**
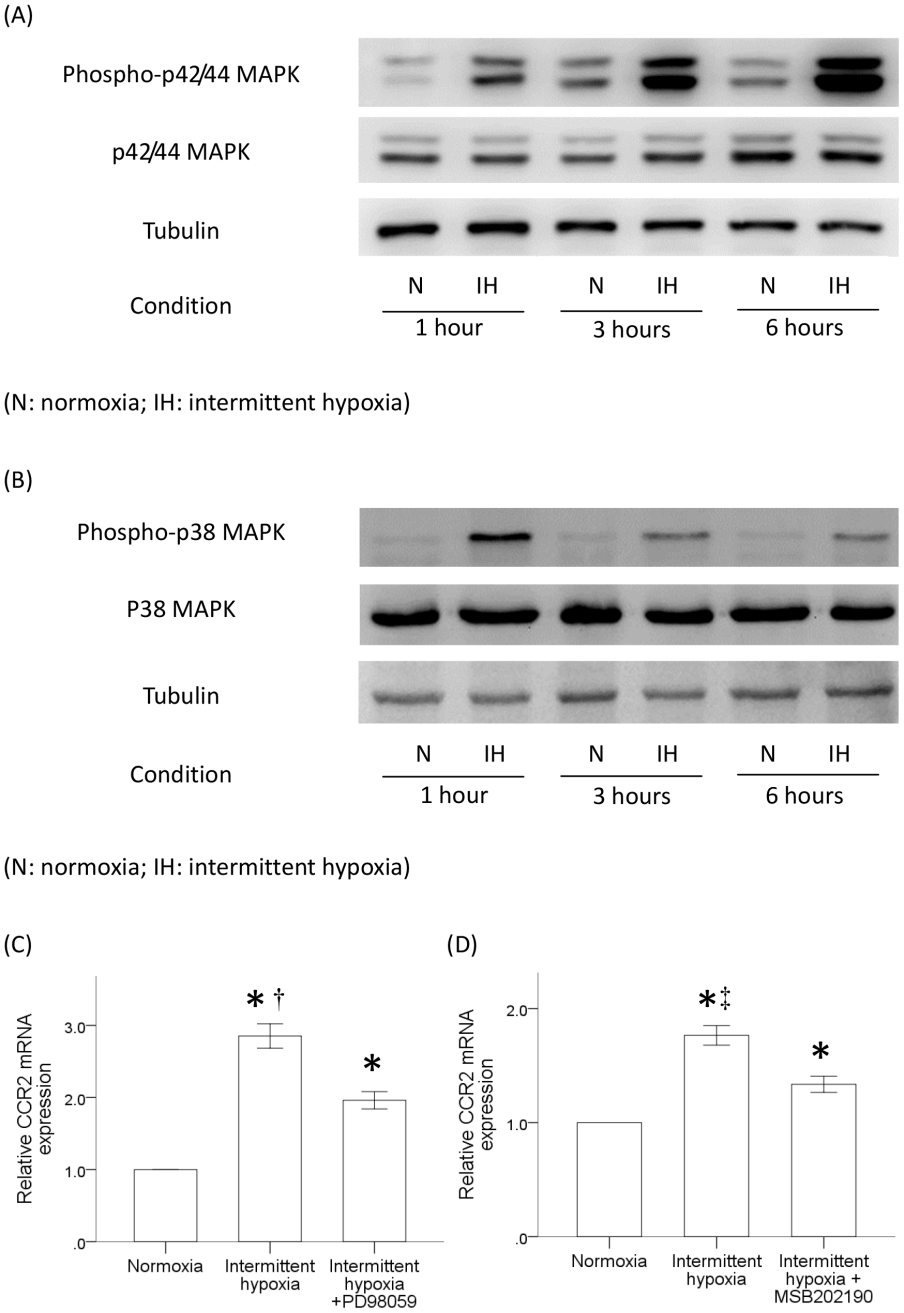
Intermittent hypoxia induced the activation of p44/42 and p38 MAPK signal pathways in THP-1 cells. THP-1 cells were treated with normoxia or intermittent hypoxia and cytosolic proteins were collected at 1, 3 and 6 hr for western blot analysis. The time-dependent activation of (A) p44/42 MAPK or (B) p38 MAPK through phosphorylation by intermittent hypoxia was investigated in THP-1 cells. Monocytes were pretreated with (C) PD98059 or (D) MSB202190 to inhibit p44/42 or p38 MAPK respectively for 1 hr and then the CCR2 mRNA expression was induced by intermittent hypoxia. (N: normoxia; IH: intermittent hypoxia) (mean ± SE, *: P<0.05 vs. Normoxia, †: P<0.05 vs Intermittent hypoxia + PD98059, ‡: P<0.05 vs Intermittent hypoxia + MSB202190).

The induction of CCR2 gene expression in monocytes has been reported to be dependent on the activation of signal pathways including ERK and p38 MAPK [26]. To further investigate the signaling pathways that might be activated by intermittent hypoxia, we performed western blot analysis to determine the phosphorylated levels of p44/42 and p38 MAPK in monocytes after treatment with intermittent hypoxia. Results demonstrated the time-dependent activation of both p44/42 and p38 MAPK in monocytes after intermittent hypoxia treatment (Figure 6A and 6B, respectively). Maximum level of phosphorylated p44/42 and p38 MAPK was found at 6 hr and 1 hr after intermittent hypoxia, respectively. Pretreatment with PD98095 and MSB202190 to inhibit p44/42 and p38 MAPK respectively in monocytes decreased the CCR2 gene expression induced by intermittent hypoxia (Figure 6C and 6D). Results demonstrated the activation of p44/42 and p38 MAPK was required for the increased CCR2 gene expression in monocytes by intermittent hypoxia.

## Discussion

Many chemokines and their receptors have been investigated and demonstrated to be responsible for the attraction, chemotaxis, adhesion and transendothelial migration of monocytes and involved in the early development of atherosclerosis [27]. Both CC and CXC chemokines are found to be expressed in human atherosclerotic plaques, and an increased expression of pro-inflammatory chemokines and their receptors correlates well with the progression of atherosclerosis within aortas of hyperlipidemic mice [28]. Studies in mice with chemokine or chemokine receptor deficient on the ApoE or LDL receptor knockout background have further confirmed their pathological roles in atherosclerosis [29]. Among the receptors, CCR2 is primarily expressed in almost all circulating monocytes, and mediates the chemotaxis of monocytes to the sites of inflammation that is involved in the pathogenesis of several inflammatory diseases [30]. Mice with deficiency of either CCL2 or CCR2 or with leukocyte CCR2-deficiency on an atherosclerotic background all showed decreased lesion formation and reduced macrophage number in the aortic root [31], [32]. The number of circulating CCR2-positive inflammatory monocytes in hypercholesterolemic animals is found to be increased [33]. In hemodialysis patients, the CCR2 expression of monocytes has been reported to positively correlate with the carotid intima-media thickness and cardio-ankle vascular index [34]. In addition to the chemotaxis of monocytes that can be induced by MCP-1 through CCR2 pathway, the activation of MCP-1-CCR2 axis in monocytes also enhances the adhesion of monocytes to vascular endothelial cells [25]. All these studies together with our novel findings that CCR2 gene expression in monocytes was up-regulated in severe OSA patients provide new evidence for the close association of OSA and cardiovascular diseases.

OSA is characterized by a cyclic occurrence of apneic events during sleep that is associated with intermittent hypoxemia and terminated by brief electroencephalographic and autonomic arousals [35]. Multiple cycles of hypoxia/reoxygenation can lead to the activation of inflammatory pathways, up-regulate the downstream expression of pro-inflammatory mediators including pro-inflammatory cytokines, chemokines and adhesion molecules, and result in the activation of various inflammatory cells, particularly lymphocytes and monocytes [36]–[38]. Except our study that demonstrated the increased CCR2 gene expression of monocytes in OSA patients and under *in vitro* condition of intermittent hypoxia, a recent study has reported the increase of CCR2 gene expression and macrophage infiltration in carotid body of rat treated with chronic intermittent hypoxia for 7 days [39]. Although the experimental conditions used in this study was different from ours, similar results all indicated that the CCR2 gene expression could be up-regulated by multiple cycles of hypoxia/reoxygenation both *in vitro* and *in vivo*. More interestingly, the monocytic CCR2 gene expression in patients of severe OSA group (AHI >30) was found to be further increased after sleep. It is likely that the condition of intermittent hypoxia during the sleep of severe OSA patients might play an important role on the increase of CCR2 expression in monocytes. As shown in our results, the increase of CCR2 expression in monocytes by intermittent hypoxia is demonstrated to be dose-dependent which might explain why the increase of CCR2 after sleep was observed only in severe OSA patients.

Growing evidence points out the importance of oxidative stress and activated inflammatory cells that play a role in the association between OSA and cardiovascular morbidity [40]. Monocytes, known to participate crucially in the entire pathological progression of atherosclerosis, have also been found to become active in OSA patients [22], [41]. In the present study, we demonstrated the increase of monocytic CCR2 expression in monocytes of severe OSA patient. Together with the previous finding that MCP-1 level is significantly high in OSA patients, indicating the increase of monocytes could be more easily attracted and adhered to endothelial cells. In addition, the expression of adhesion molecules such as CD15 and CD11c, adhesion index and ROS in monocytes were also found to be up-regulated in OSA patients compared to control [42]. Indeed, monocytes isolated from OSA patients appeared to acquire increased adhesive activity to endothelial cells [43], [44]. Circulating monocytes adhere to the endothelium, then transmigrate into the subendothelium, and subsequently invade the matrix of the intima during differentiation toward macrophages, which plays an important role in the early stage of atherosclerosis [45]. The number of macrophages is a lot more abundant in atherectomy materials from unstable angina and abdominal aortic aneurysm [46], [47]. The activation and infiltration of monocytes not only directly participates in the formation of atherosclerotic plaque, but is also associated with the increased risk of plaque rupture [48], [49].

It is interesting for our study to demonstrate the increase of CCR2 gene expression in monocytes of severe OSA patients, however, some limitations still need been to be discussed in this study. Firstly, the small sample size was possibly due to the limited number of patients and stringent inclusion criteria. During patient recruitment, we have excluded the possible confounders that could influence the CCR2 expression such as ischemic heart disease, hypertension, diabetes, hyperlipidemia, cerebrovascular disease or renal disease. In addition, this study was carried out using the monocytes purified from each patient that made the study more difficult. Initially, 72 patients have enrolled in this study, but 18 patients were excluded to minimize the effects from other potential confounders. Based on our experience and also results published in previous studies comparing patients with normal control, BMI is often found to be a critical factor affecting OSA. In our study, both AHI and ODI in 54 patients were significantly different among four groups (AHI ≦5, 5< AHI ≦15, 15< AHI ≦30, and AHI >30). The potential influence by BMI was excluded because the BMI value among four groups did not differ significantly. Since this study aimed to understand the potential effect of intermittent hypoxia, patients with AHI ≦5 were considered control in our study.

Instead of average total sleep time, we did measure the sleep efficiency as shown in Table 1 and the results had no statistical significance. As for the duration of OSA, since the record from each patient was considered not scientifically reliable, we did not add the data in our study. Results obtained from our *in vitro* cell study, though under the simplified condition, provided the first evidence that the CCR2 expression in monocytes was indeed up-regulated by intermittent hypoxia. Our results, for the first time, demonstrated that the induction level of CCR2 mRNA expression in monocytes was higher when cells treated with further hypoxia, providing a reason for the increase of CCR2 gene expression in severe OSA patient. Although we did not directly compare the response to intermittent hypoxia using monocytes from OSA patients with different severity, we tested the response of monocytes *in vitro* under condition that partially resembled the severe OSA. We found that in the presence of TNF-α or CRP, two inflammatory markers known to be increased in severe OSA patients, the CCR2 mRNA expression could be further enhanced at least 75% by intermittent hypoxia, suggesting the intermittent hypoxia-induced CCR2 mRNA expression in monocytes could further be up-regulated in the presence of other factors involved in severe OSA.

Finally, we divided recruited patients into 4 groups according to the standard OSA criteria, include the relative normal subjects who's AHI <5 as controls. It's not easy to find those patients with the same high body mass index but no any respiratory event during sleep. Thus, lack of normal controls is another limitation in our study.

In summary, this study, for the first time, demonstrated the increase of CCR2 gene expression in monocytes of severe OSA patients. Intermittent hypoxia, the hallmark of obstructive sleep apnea, was proved to increase the CCR2 gene expression and the chemotaxic ability of monocytes toward MCP-1. Intermittent hypoxia was also found to further enhance the adhesion of monocytes to vascular endothelial cells. Both ERK and p38 MAPK were confirmed to be involved in the signaling pathway for the induction of CCR2 in monocytes by intermittent hypoxia. These findings may shed some light on mechanisms involved in increased monocyte chemotaxis and adhesion under IH conditions that may lead to the development of atherosclerosis in patients with OSA. These results also strongly suggest an important role of CCR2, therefore, to reduce CCR2 expression or to block its function in monocytes by various antagonists could be a promising strategy to prevent atherosclerosis in patients with OSA.
